# Peer Review of “Investigating the Variable Component of the Systematic Error, a Neglected Error Parameter: Theoretical Reevaluation Study”

**DOI:** 10.2196/90221

**Published:** 2026-02-27

**Authors:** Anonymous

**Keywords:** repeatability condition, reproducibility within laboratory condition, measurement, systematic error, clinical laboratory, quality control, bias, QC, statistical, statistics, mathematics, computer simulation, standard deviation

*This is the peer-review report for “Investigating the Variable Component of the Systematic Error, a Neglected Error Parameter: Theoretical Reevaluation Study.”*

## Round 1 Review

### General Comments

With an emphasis on the idea of VCSE(t) (variation in control sample error over time), the study [1] provides a thorough examination of variation in laboratory data. The study emphasizes the significance of differentiating between random and systematic errors, suggests fresh approaches for precisely calculating sVCSE (an SD), and supports updated quality control procedures.

### Specific Comments

#### Major Comments

1. The study does not provide empirical confirmation of suggested approaches using real-world data, although it mentions computer simulations and experimental verification. The suggested methodologies’ efficacy and dependability are yet unknown in the absence of empirical validation.
2. Linear drifts in daily means across time are assumed in the study. Numerous factors, such as the environment, instrument calibration, and reagent stability, can affect real-world drift patterns and lead to nonlinear trends in daily means over time. The study might have simplified the complicated nature of drift processes by assuming linearity, which could result in estimates of mean values and error components that are not true.
3. The assumption that information from internal quality control sources alone can be used to accurately calculate VCSE(t) is inaccurate. Even though internal quality control data offer insightful information on short-term variability, they might not include all sources of variation, particularly those pertaining to outside variables like shifts in the environment, instrument performance, or operator technique. Ignoring these outside influences could result in an inaccurate or understated VCSE(t), which would compromise the validity of the suggested quality control techniques.

#### Minor Comments

1. Although there is a suggestion in the Conclusions section that the present quality control paradigm needs to be revised, there is no concrete plan or set of recommendations based on statistical or mathematical concepts.
2. The paper lacks a Discussion section, which could have allowed the author to interpret and contextualize the study’s findings. Additionally, it could have provided an opportunity to compare the study’s findings with previous studies, discuss their implications, and address potential sources of error or bias.

## Abbreviations

VCSE(t): variation in control sample error over time
